# Induced responses to the wheat pathogen: Tan Spot—(*Pyrenophora tritici-repentis*) in wheat (*Triticum aestivum*) focus on changes in defence associated and sugar metabolism

**DOI:** 10.1007/s11306-023-02084-w

**Published:** 2024-01-31

**Authors:** Larissa Carvalho Ferreira, Flavio Martins Santana, Sandra Maria Mansur Scagliusi, Manfred Beckmann, Luis A. J. Mur

**Affiliations:** 1 Department of Life Sciences, Aberystwyth University, Aberystwyth, UK; 2 Everglades Research and Education Center, University of Florida, Belle Glade, FL USA; 3 Embrapa Trigo, Passo Fundo, RS Brazil

**Keywords:** Tan Spot disease, *Pyrenophora tritici-repentis*, Wheat, Flow-infusion electrospray mass spectrometry, Plant defence

## Abstract

**Introduction:**

Tan Spot (TS) disease of wheat is caused by *Pyrenophora tritici-repentis* (*Ptr*), where most of the yield loss is linked to diseased flag leaves. As there are no fully resistant cultivars available, elucidating the responses of wheat to *Ptr* could inform the derivation of new resistant genotypes.

**Objectives:**

The study aimed to characterise the flag-leaf metabolomes of two spring wheat cultivars (*Triticum aestivum* L. cv. PF 080719 [PF] and cv. Fundacep Horizonte [FH]) following challenge with *Ptr* to gain insights into TS disease development.

**Methods:**

PF and FH plants were inoculated with a *Ptr* strain that produces the necrotrophic toxin ToxA. The metabolic changes in flag leaves following challenge (24, 48, 72, and 96 h post-inoculation [hpi]) with *Ptr* were investigated using untargeted flow infusion ionisation-high resolution mass spectroscopy (FIE-HRMS).

**Results:**

Both cultivars were susceptible to *Ptr* at the flag-leaf stage. Comparisons of *Ptr*- and mock-inoculated plants indicated that a major metabolic shift occurred at 24 hpi in FH, and at 48 hpi in PF. Although most altered metabolites were genotype specific, they were linked to common pathways; phenylpropanoid and flavonoid metabolism. Alterations in sugar metabolism as well as in glycolysis and glucogenesis pathways were also observed. Pathway enrichment analysis suggested that *Ptr*-triggered alterations in chloroplast and photosynthetic machinery in both cultivars, especially in FH at 96 hpi. In a wheat-*Ptr* interactome in integrative network analysis, “flavone and flavonol biosynthesis” and “starch and sucrose metabolism” were targeted as the key metabolic processes underlying PF–FH–*Ptr* interactions.

**Conclusion:**

These observations suggest the potential importance of flavone and flavonol biosynthesis as well as bioenergetic shifts in susceptibility to *Ptr*. This work highlights the value of metabolomic approaches to provide novel insights into wheat pathosystems.

**Supplementary Information:**

The online version contains supplementary material available at 10.1007/s11306-023-02084-w.

## Introduction

Tan Spot (TS) disease of wheat (syn. yellow Spot), caused by *Pyrenophora tritici-repentis* (*Ptr*), can lead to yield losses of up to 60% (Rees & Platz, 1989). Most of the yield loss is attributed to TS disease development on the flag leaves, leading to smaller grain sizes (Bhathal et al., 2003; Rees & Platz, 1989). *Ptr* is a necrotrophic pathogen where different strains can produce one or more phytotoxic effectors, (ToxA, ToxB, and ToxC) that can induce necrosis and chlorosis in sensitive lines (Effertz et al., 2002; Sarma et al., 2005; Strelkov et al., 1999). Such necrotrophic mechanisms can also involve the elicitation of host programmed cell death (PCD) (Kariyawasam et al., 2023) although this has not been clearly established for TS. *Ptr* strains are classified into different races based on the production, either singly or in combination, of these toxins (Lamari et al., 1995). Worldwide, ToxA-producing *Ptr* races are the most common (Abdullah et al., 2017; Ali & Francl, 2003; Gamba et al., 2012; Kokhmetova et al., 2020; Lamari et al., 2003). ToxA is a proteinaceous necrotising effector that is internalised to chloroplasts and binds to the ToxABP1 protein (Manning et al., 2007, 2008) to compromise photosynthesis (Abeysekara et al., 2010; Effertz et al., 2002; Friesen & Faris, 2004). Some quantitative trait loci (QTL) for TS resistance have been defined and designated as *Tsr* (*Tan spot resistance*) loci (Anderson et al., 1999; Chu et al., 2008, 2010; Faris & Friesen, 2005; Faris et al., 1996, 2013; Gamba & Lamari, 1999; Hu et al., 2019; Li et al., 2011; Singh et al., 2008, 2010, 2016; Tadesse et al., 2006a, 2006b). Eight major *Tsr* genes (*Tsrl*, *Tsr2*, *Tsr3*, *Tsr4*, *Tsr5*, *Tsr6*, *TsrHar*, and *TsrAri*) have been mapped to different points on the wheat genome (McIntosh et al., 2013).

The development of resistant germplasm could be improved if the underlying mechanism(s) of TS disease development were more fully understood. This is particularly the case with metabolomic assessments, and no such study has been undertaken on TS on wheat. Recent advancements in mass spectrometry technologies have enabled high throughput quantitative and qualitative assessments of metabolic compounds, including from plants and microorganisms (Allwood et al., 2021). As a result, metabolomics has become an important technology for plant breeders and phytopathologists, especially when integrated with systems biology tools (Aliferis et al., 2014; Kumar et al., 2017; Rosato et al., 2018; Weckwerth, 2003). More specifically, metabolomics has been employed in other wheat–pathogen interactions to deliver new insights. For example, metabolomics has been used to characterise responses to *Bipolaris sorokiniana* (Ye et al., 2019), or *Puccinia striiformis* f. sp. *tritici*, the cause of yellow rust (Mashabela et al., 2023). Some metabolomic changes have been linked to defensive responses in wheat, for example, against *Fusarium graminearum* (Gauthier et al., 2015), and stem rust caused by the pathogen *Puccinia graminis* f. sp. *tritici* (Maserumule et al., 2023). Equally, metabolomic changes can suggest the mechanisms of “systemic induced susceptibility” by *Zymoseptoria tritici* (Seybold et al., 2020) or how the toxin deoxynivalenol (DON) contributes to *F. graminearum* caused Fusarium head blight. Defensive responses are often linked to changes in phenylpropanoid metabolism which feeds into flavonoid biosynthesis well as hydroxycinnamic acid and monolignol production which could reinforce cell walls (Allwood et al., 2021; Maserumule et al., 2023; Seybold et al., 2020). Changes in the levels of phytoanticipins such as benzoxazinoids have also been noted (Mashabela et al., 2023; Seybold et al., 2020). Other metabolomic changes are consistent with bioenergetic changes in for example, the tri-carbonic acid (TCA) cycle and γ-aminobutyric acid (GABA) shunt and wide impacts on primary metabolism on such as amino acid and lipid processing (Mashabela et al., 2023). The power of metabolomics is such that it can also identify key signalling components in pathogenesis; for example, sphingolipids influencing appressorial development in *Magnaporthe oryzae* (Liu et al., 2019).

In this study, we focused on two cultivars emerging from a Brazilian-based wheat breeding programme. Fundacep Horizonte (FH) (Cooperativa Central Gaúcha Ltda Tecnologia/FUNDACEP) and PF 080719 (PF), the latter showing some evidence of resistance to TS following field assessments (Cunha et al., 2016). FH is a high-yielding spring wheat cultivar widely grown in southern Brazil. PF is also a highly productive variety with significant resistance to wheat leaf rust, powdery mildew, and leaf blotch. We characterised the metabolomic responses of PF and FH to *Ptr*. Flag leaves provide carbon assimilates to the grain as it develops (Evans & Rawson, 1970) and so these are vital to good grain yields in cereals (Carmo-Silva et al., 2017; Khaliq et al., 2008; Wazziki et al., 2015). Photosynthetic performance in flag leaves is influenced by genetic background, the use of fertilizers as well as abiotic and biotic stresses (Evans, 1983; Guóth et al., 2009; Inoue et al., 2004; Wazziki et al., 2015). Therefore, the ability to sustain carbon assimilation activity during such as *Ptr* infection could be a valuable trait for wheat breeding (Araus et al., 2002; Carmo-Silva et al., 2017; Yang & Luo, 2021).

Focusing on flag leaves, we used untargeted metabolomics approach allowed the detection of key metabolites and pathways underlying the responses of each cultivar to *Ptr* infection as well as the genotypic differences between them. We observed mostly different individual metabolite changes in PF and FH, but many could be mapped to common pathways particularly, “flavone and flavonol biosynthesis” and “starch and sucrose metabolism”. This suggest were targeted as the metabolomic changes linked to ultimately failed defences and sugar/bioenergetic changes.

## Material and methods

### Host classifications based on lesion scores

Seeds from *Triticum aestivum* cv. PF and cv. FH were sown in John Innes No2 compost and grown in controlled-environment growth chambers (Conviron, UK) under a 16 h light period at 21 °C and 8 h of darkness at 18 °C. The cultivars were either mock-inoculated (M) or inoculated with *Ptr* (I). Each treatment was applied to three replicates (25 cm diameter pots × 20 cm high pots) that were grown in a randomised manner within the growth chamber. The treatments were applied at either of two growth stages: GS13 (three-leaf seedlings) and GS65 (flowering/anthesis).

### *P. tritici-repentis* infections

The *Ptr* strains (BR13, BR29 and BR154) used in this study were isolated from naturally infected wheat fields in southern Brazil. Mycelial plugs of *Ptr* cultures were transferred to Petri dishes containing V8 media (agar = 15 g; CaCO_3_ = 3 g; V8-Juice = 150 mL, dH_2_O = 850 mL). The plates were sealed and incubated upside-down for 5 days under continuous darkness at 25 °C. Following the protocol of Lamari and Bernier (1989), the Petri dishes were flooded with sterilised ultrapure water and the mycelia were flattened with the bottom of a sterile test tube. The resulting suspension was discarded, and the plates were incubated at 25 °C for 24 h in constant light, followed by 24 h in total darkness at 15 °C. The mycelia were displaced using a paintbrush into a solution of ultrapure water with 0.5% (v/v) Tween 80. The mycelial suspension was then sieved and vortexed to aid conidial detachment. Conidia concentrations were adjusted to 3000 conidia/mL with ultrapure water with 0.5% (v/v) Tween 80. Plants sprayed with to run-off with the conidial suspensions. Inoculated plants were maintained at ~70% humidity in a humidity chamber for 24 h. Mock-inoculated plants were treated only with sterile ultrapure water with 0.5% (v/v) Tween 80. Lesion phenotypes were scored as R = resistant; MR = moderately resistant; MRMS = moderately resistant to moderately susceptible; MS = moderately susceptible; S = susceptible.

### Metabolite extractions

Disks (1 cm diameter) (n = 5 discs per biological replicate; i.e., “pot”) were punched out from fully extended flag leaves from each genotype at 0, 24, 48, 72, 96, and 168 h post-inoculation with the *Ptr*. The samples were immediately frozen in liquid nitrogen and stored at −80 °C until processed. Samples of 40 mg (±1 mg) of leaf tissue was placed in 2 mL sterile microcentrifuge tubes, each containing one acetone-cleaned stainless-steel bead. The samples were flash-frozen in liquid N_2_ and homogenised using a ball mill system. Then, 1 mL of chloroform:methanol:dH_2_O (1:2.5:1) solution was added to each sample, followed by incubation in a shaker at 4 °C for 15 min. The samples were centrifuged at 5000 × g for 5 min and the supernatant was transferred to a new microcentrifuge tube from which 100 μL was transferred to a glass vial and sealed. Untargeted metabolite fingerprinting was performed by Flow Infusion Electrospray Ionization High-Resolution Mass Spectrometry (FIE-HRMS), where the mass-to-charge ratio features (*m/z*) were generated in negative and positive ionisation modes using a Q Exactive hybrid quadrupole-Orbitrap mass spectrometer (Thermo-Scientific, UK). Two, 20 μL, injections were performed for each sample as technical replicates.

### Metabolomic data processing and analysis

The raw data was filtered based on the relative standard deviation of 0.5 and a minimum occupancy of 2/3 in each class using the R package metabolyseR version 0.14.6. The *m/z* data was normalised based on total ion count (TIC) normalisation and visualised using unsupervised principal component analysis (PCA). Differentially accumulated metabolites (DAMs) were identified in each genotype based on pairwise comparisons between mock- and *Ptr*-inoculated samples within each time point. Datasets were designated as PF24, PF48, PF72, PF96, FH24, FH48, FH72, and FH96 to reflect genotype and timepoints. Samples collected before treatment were compared between both genotype and the outputs designated as PF/FH. Two-sided Welch t-tests were performed in all these comparisons and the features with adjusted *P*-values < 0.05 after Bonferroni correction were considered significant DAMs. The peak intensity data was summarised by mean in each subset of genotype/treatment/hpi after log_2_(x + 1) transformation. Fold changes were then calculated between inoculated (I) over the mock-treated samples, (M) (i.e., (I − M)/M).

Molecular formula were assigned to the explanatory features using the R package MFassign v0.7.7. Functional enrichment was performed by mapping the assigned features to KEGG metabolic network using hypergeometric, PageRank and Diffusion methods within the FELLA package (Kanehisa et al., 2012; Picart-Armada et al., 2018), using *Aegilops tauschii* as the reference metabolome. To extract a significant sub-network, the diffusion method was used to score the enriched nodes. After normalisation through z-scores, the nodes with *P*-value < 0.05 were selected. The resulting graph was imported to Cytoscape v 3.8.2 (Shannon et al., 2003) for visualisation.

## Results

### Phenotypic characterisation of *Ptr* interactions with FH and PF

**Fig. 1:**
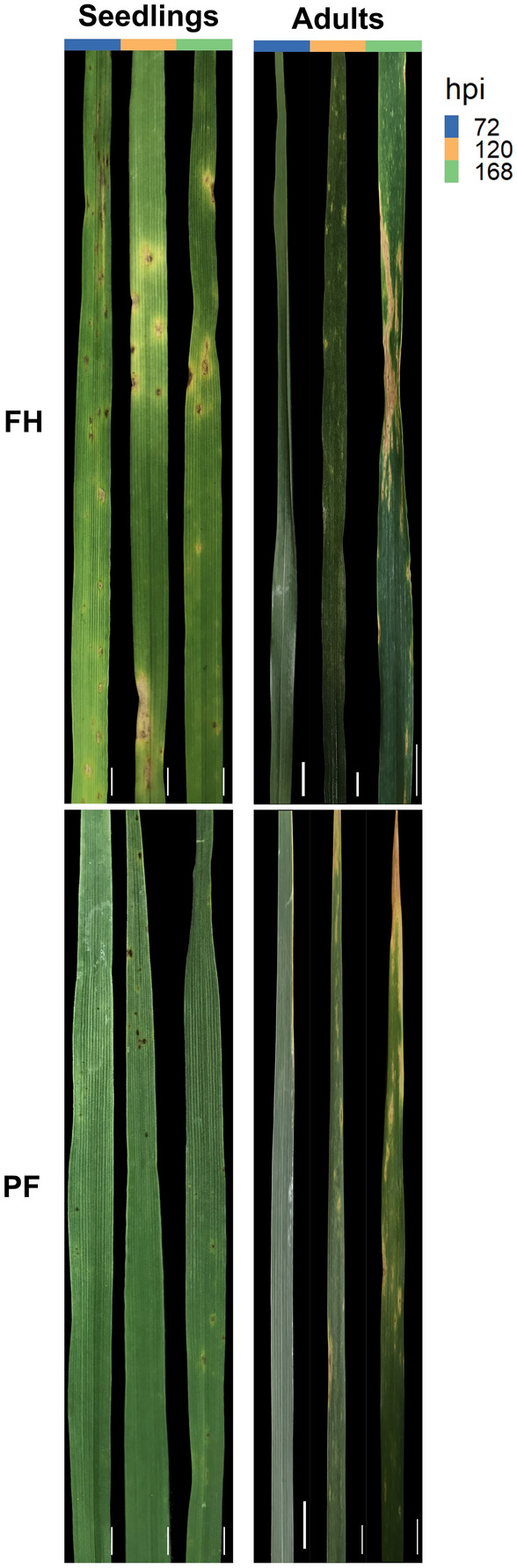
Tan Spot symptoms on leaves from PF 080719 (PF) and Fundacep Horizonte (FH) wheat cultivars challenged with *P. tritici-repentis* (*Ptr*) *strains* BR13, BR29 and BR154. Images of representative leaves at 72, 120 and 168 h post inoculation (hpi) with *Ptr.* Scale bar = 1 cm

We first evaluated responses to *Ptr* challenge in FH and PF at two different developmental stages; the seedling stage (GS13) and during flowering, when the flag leaf had fully emerged (GS65). Plants were inoculated with three ToxA positive strains with varying levels of virulence (BR13 = mild; BR154 = moderate virulence; BR29 = highly virulent), in two independent experiments. Scoring for disease severity indicated that response in PF widely varied from resistant to susceptible dependent on the *Ptr* virulence and growth stage (Table 1; Fig. 1).

**Table 1:**
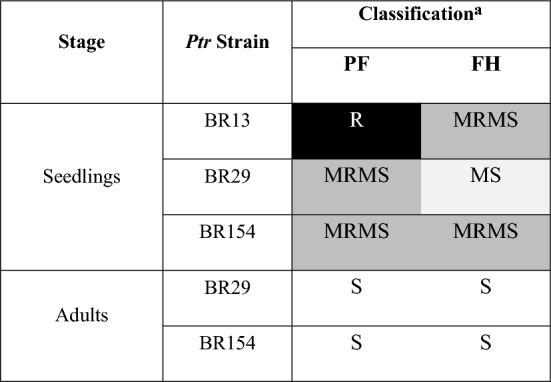
Classification of disease scores against *P. tritici-repentis* infection in the wheat lines PF 080719 (PF) and Fundacep Horizonte (FH) at different growth stages

*R* resistant, *MR* moderately resistant, *MRMS* moderately resistant to moderately susceptible, *MS *moderately susceptible, *S* susceptible

^a^Based on mean scores from assessments at 336 h post inoculation (hpi)

At the seedling stage PF exhibited a resistant phenotype when challenged with the BR13 but was only moderated tolerant to moderately susceptible (MRMS to MS) when infecting with either BR154 or BR29. Adult PF plants were highly susceptible to BR29. FH seedlings exhibited MRMS to MS phenotypes, but adult plants were clearly susceptible to all strains of *Ptr*. PF had exhibited minimal chlorosis following interaction with *Ptr* by 168 hpi in seedlings (Fig. 1). At the adult stage, chlorosis was observed in PF from 120 hpi. In FH, both chlorosis and coalescence of the fleck-like lesions were observed from 120 hpi when infecting at either growth stage (Fig. 1).

### Metabolomic characterisation of *Ptr* interactions with FH and PF

**Fig. 2:**
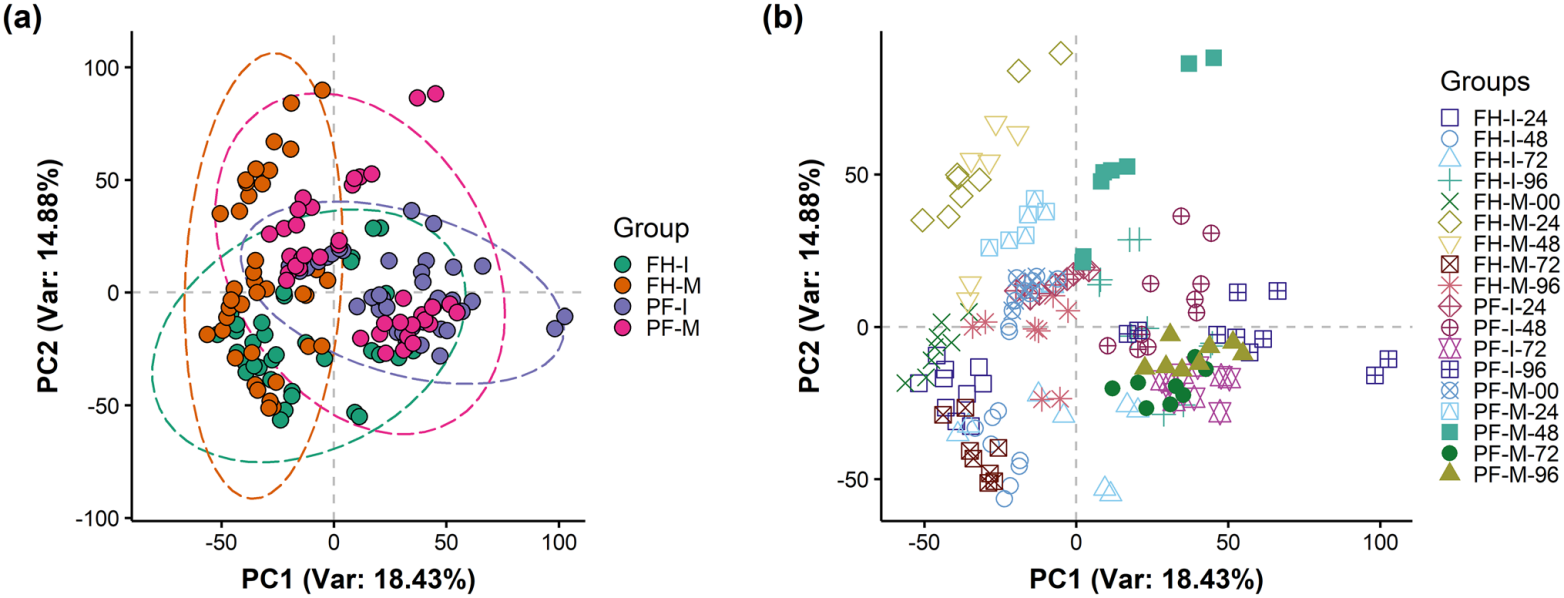
Principal component analysis of the metabolomes (**a**) mock-treated (M) versus leaves infected with *P. tritici-repentis* strain BR154 (I) from the wheat lines PF 080719 (PF) and Fundacep Horizonte (FH). Also (**b**), comparing PF and FH at 0, 24, 48, 72 and 96 h post inoculation

Given the importance of the wheat flag leaf, metabolomic assessments of *Ptr* inoculation focused on that stage. Flag leaf samples were collected from FH and PF at 0, 24, 48, 72, and 96 h post inoculation (hpi) with *Ptr* strain BR154. Samples were extracted and profiled using FIE-HRMS and the derived data were assessed by PCA. The major sources of variation (18.43% of the variance) across the first principal component (PC1) discriminated between genotype (FH vs. PF) (Fig. 2a). There were no significant differences between mock and inoculated samples when all time points were considered together. When different time points were considered, the different sampling times did show discrete clustering. Interestingly, samples from mock-inoculated (M) plants exhibited a greater variation compared to inoculated (I) samples in both genotypes (Fig. 2b).

**Fig. 3:**
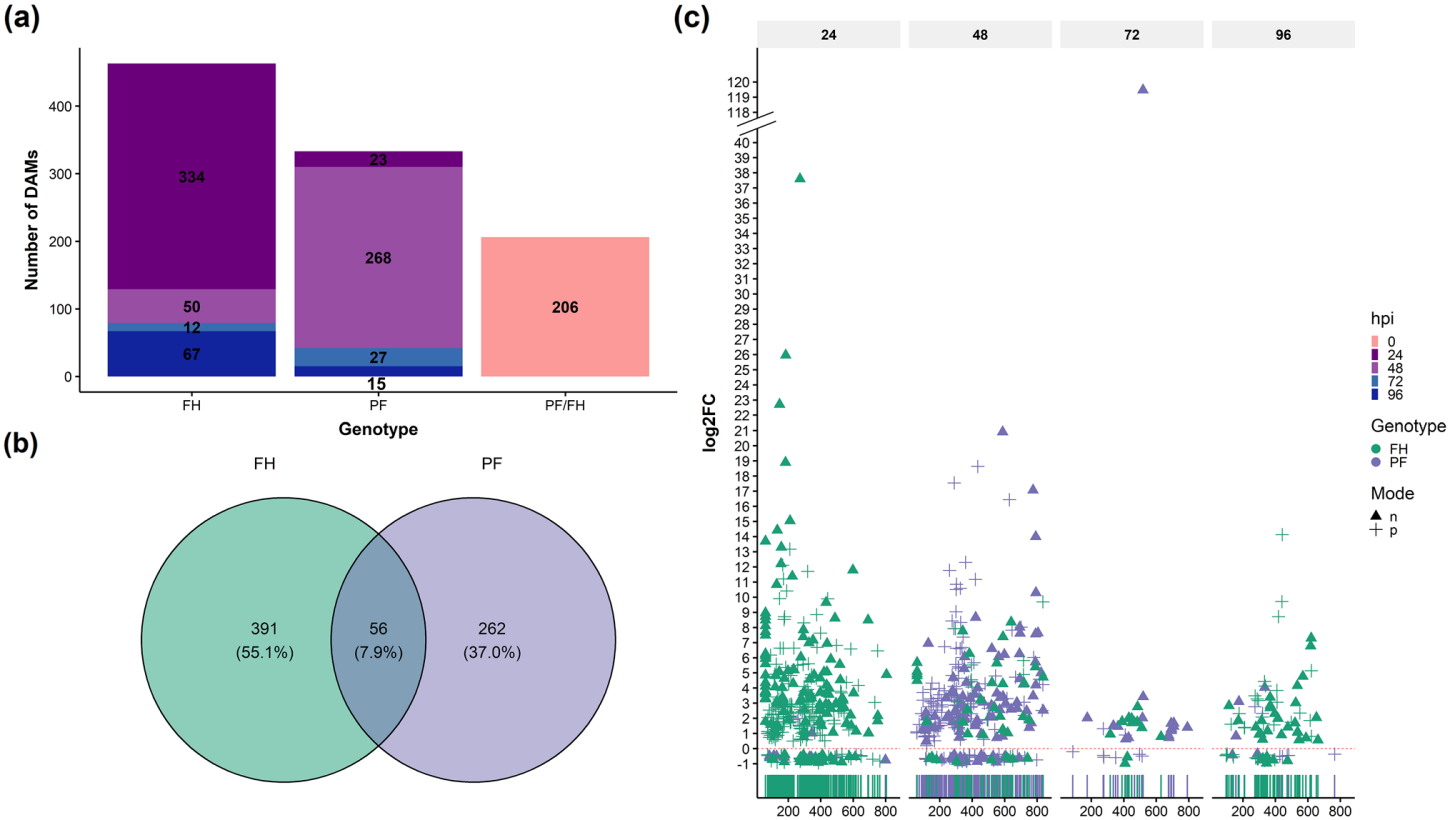
Metabolomics comparisons between the wheat lines PF 080719 (PF) and Fundacep Horizonte (FH) challenged with *P. tritici-repentis* strain BR154. **a** Number of differentially accumulated metabolites (*P* < 0.05) in mock-treated (M) versus infected (I) samples in each line and also comparing basic genotypic differences between unchallenged PF and FH (PF/FH). **b** Venn diagram displaying number of common and unique differentially accumulated mass-ions (DAMs) in PF and FH. **c** Scatter-rugged plot showing DAMs differentiating between ionisation mode (negative/positive) and log_2_(fold change) between I and M at 24, 48, 72 and 96 hpi

Given the minor metabolomic changes seen with *Ptr* challenge, pairwise comparisons between I versus M samples from each line in each time point were performed using Welch’s two-sided t-test to identify differentially accumulated mass-ions (DAMs). A total of 1002 DAMs were identified (Fig. 3; Table S1).

The significant differences seen between PF and FH at 0 h (206 DAMs) were taken as indicating innate genotypic differences. Most of the explanatory features underlying genotypic differences between PF and FH were present at higher levels in PF, with log_2_ fold changes up to 34.85 (Table S1). Of these DAMs, approximately 80% are products from the negative ionisation reaction. Considering DAMs that could be identified, these indicated different levels of hippurate, diacetyl, acetone cyanohydrin, and the flavonoids dihydrophaseic acid (DPA), desulfoglucotropeolin (UGT4B1), 3,7-Di-*O*-methylquercetin, apigenin 7-*O*-[beta-d-apiosyl-(1→2)-beta-d-glucoside], and 1-*O*-vanilloyl-beta-d-glucose (Table S1). Two mass ions annotated as the flavonoids apigenin 7-*O*-neohesperidoside (*m/z* = 615.14032, negative mode) and vitexin (*m/z* = 431.09912 and 432.1022, negative mode) were the only compounds that could be annotated that were found at higher levels in FH compared to PF (log_2_FC values between 0.75 and 0.89).

We next considered how many of the *Ptr*-induced changes in each genotype. Following infection, FH had the most DAMs at 24 hpi, which accounted for 72% of the total for this genotype. Conversely, PF showed most DAMs at 48 hpi compared to the M samples (Fig. 3a). Only 56 DAMs were seen in both PF and FH following challenge to *Ptr* (Fig. 3b; Table 2). Of these, *Ptr* infection significantly increased the levels of 53 metabolites, a decrease in one compound in both genotypes, but two metabolites had opposite accumulation patterns in PF and FH (Table 2).

**Table 2:** Common differentially accumulated metabolites (DAM) found in pairwise comparisons between *Ptr*-inoculated samples FH and PF at 24, 48, 72, and 96 h post-inoculation and their respective uninoculated controls

*m/z*^a^	FH24	FH48	FH72	FH96	PF24	PF48	PF72	PF96	MF	Isotope	Adduct
p359.12054	2.07					12.30			C_14_H_20_N_3_O_6_P	13C	[M + H]^1+^
p419.1235				8.71		11.17			C_16_H_26_O_10_	13C2	[M + K]^1+^
n805.33246	4.89					7.64			C_37_H_60_O_15_P_2_		[M − H]^1−^
n129.0379	10.84					6.95			C_6_H_9_OP	13C2	[M − H]^1−^
p325.03995	2.94					6.60			C_16_H_15_O_3_P		[M + K]^1+^
n598.3089	11.79					6.21			C_28_H_50_O_11_	13C	[M + Cl]^1−^
p338.1908				4.14		6.02			C_14_H_29_N_2_O_5_P	13C	[M + H]^1+^
n789.31079		5.39				5.72			C_36_H_55_O_17_P		[M − H]^1−^
p543.24054	2.35					4.80			C_23_H_38_N_2_O_11_	18O	[M + Na]^1+^
p316.09979	2.73					4.65			C_9_H_13_N_7_O_6_		[M + H]^1+^
p835.40015		9.69				4.22			C_38_H_60_NO_16_P		[M + NH_4_]^1+^
n433.19092	9.65					4.00			C_21_H_32_O_7_	13C2	[M + Cl]^1−^
p196.05597	2.33					3.97			C_5_H_11_N_2_O_4_P	13C	[M + H]^1+^
p442.1196				14.13		3.96					
p302.10339	2.30				1.03	3.94			C_15_H_13_N_3_O_4_	13C2	[M + H]^1+^
p143.07237	3.28					3.90					
p410.24127				3.06		3.63			C_19_H_31_N_5_O_5_		[M + H]^1+^
p295.168		7.91		3.18		3.56			C_12_H_25_N_2_O_4_P	13C2	[M + H]^1+^
p197.05678	2.47					3.55			C_5_H_11_N_2_O_4_P	18O	[M + H]^1+^
p373.06198	4.64					3.35					
p210.07156	13.17					3.18					
p409.22824				3.85		2.96	0.90		C_16_H_32_N_4_O_8_		[M + H]^1+^
p346.04242	2.13					2.95					
n518.18787	5.55					2.85			C_22_H_33_NO_13_		[M − H]^1−^
p394.24567		4.55				2.77			C_17_H_36_N_3_O_5_P		[M + H]^1+^
n535.2688				4.16		2.74			C_26_H_44_O_9_		[M + Cl]^1−^
p614.22638				1.86		2.73					
n640.11554		8.35				2.59			C_26_H_27_NO_18_		[M − H]^1−^
n569.29651				4.77		2.56			C_34_H_44_O_5_	13C2	[M + Cl]^1−^
p178.04535	8.71					2.55					
p212.03348	1.71					2.39			C_5_H_8_NO_3_PS	13C	[M + NH_4_]^1+^
n114.03838				2.83		2.36			C_5_H_8_NP	13C2	[M − H]^1−^
p395.24908		5.52				2.30			C_17_H_36_N_3_O_5_P	13C	[M + H]^1+^
n525.30792				3.02		2.07			C_28_H_46_O_9_		[M − H]^1−^
p300.14139	2.48					2.05			C_9_H_21_N_3_O_8_		[M + H]^1+^
p391.21811				1.56		1.97			C_19_H_33_N_3_O_3_	13C	[M + K]^1+^
p497.14703				1.74		1.88			C_23_H_30_O_8_P_2_		[M + H]^1+^
n432.18832	2.95					1.82			C_21_H_32_O_7_	13C	[M + Cl]^1−^
n280.02603	3.30					1.76					
p440.25189				9.71		1.71			C_43_H_74_O_18_		[M + 2H]^2+^
p201.07129	5.07					1.70					
n420.15143	5.05					1.69					
p308.18015		3.08				1.66			C_14_H_24_O_4_S	13C2	[M + NH_4_]^1+^
p132.08009	1.19					1.56					
p171.06078	2.03					1.49			C_3_H_10_N_2_O_6_		[M + H]^1+^
p111.03996	1.69					1.49					
p177.05382				2.32		1.41			C_5_H_13_O_3_P	18O	[M + Na]^1+^
p130.04926	1.41					0.86			C_6_H_9_OP	13C	[M + H]^1+^
p137.05547	0.89					0.76			C_4_H_10_NO_2_P	13C	[M + H]^1+^
n551.19818		5.64				0.72			C_25_H_40_O_7_P_2_	18O	[M + Cl]^1−^
p326.05203				1.66		0.60			C_7_H_18_N_3_O_7_P		[M + K]^1+^
p168.04987	1.45					0.51			C_9_H_20_NO_10_P	13C	[M + 2H]^2+^
p337.8598	−0.60					−0.83			C_9_H_2_NO_3_P_3_S		[M + K41]^1+^
n339.8223	3.53							−0.73			[M − 3H]^3−^
n467.15039		0.90					1.71		C_20_H_30_O_10_		[M + Cl37]^1−^
p274.12589	−0.62						1.31	2.77	C_15_H_16_N_2_O3	13C	[M + H]^1+^

Numbered cells represent the relative log_2_ fold change values from each significant comparison, whereas blank cells are non-significant interactions

*MF *molecular formula

^a^The prefix letters indicate the ionisation modes: n = negative, p = positive

Following DAM annotation, *Ptr* was seen to induce increases in *S*,*S*-dimethyl-beta-propiothetin, piperideine, umbelliferone, phytosphingosine, but there was a lowering in the levels of anthranilate, l-valine, and *N*-methylethanolamine phosphate at 24 hpi in FH. At 72 hpi, levels of the flavonoids quercitrin, vitexin, and vitexin 2ʺ-*O*-beta-d-glucoside were significantly increased in flag leaves infected with *Ptr*. By 96 hpi, there was the downregulation of 6-deoxydihydrokalafungin (DDHK), an intermediate in the biosynthesis of type II polyketide products. With PF there was an accumulation of phenylpropanoids and flavonols (e.g., coumarin and 1-*O*-sinapoyl-beta-d-glucose), but at only 48 hpi which represented a quicker response than seen with FH. PF also saw, there was an accumulation of glucosinolates at 48 hpi. Wider changes at 48 hpi in PF included d-glucosaminate, xanthohumol, DDHK, l-homomethionine, 3-*O*-alpha-mycarosylerythronolide B and l-valine (Table S1). PF samples from 48 hpi also saw significant decreases in sucrose in the infected flag leaves. Strikingly, phosphatidylinositol diphosphate (*m/z* = 339.8223, negative mode [M − 3H]^3−^), exhibited a × 11.5-fold increase in inoculated flag leaves from FH at 24 hpi compared the controls, but it was significantly downregulated in PF at 96 hpi (fold change of × 1.66). A converse pattern was seen with metabolite with the predicted molecular formula C_15_H_16_N_2_O_3_ (*m/z* = 274.12589, positive mode), showed significant increases in PF at 72 and 96 hpi but was suppressed at 24 hpi in FH compared to controls (Table 2).

### Insights into responses to *Ptr* through pathway enrichment and network analysis

To provide an overview of key metabolic changes occurring following *Ptr* infection, pathway enrichment, and integrative network analyses were performed. The explanatory features identified by our pairwise statistical analysis were mapped to KEGG Compound entries and screened against the KEGG metabolic network using PageRank, hypergeometric, and diffusion algorithms. The over-representation analysis targeted seven significantly enriched pathways at different times and between genotypes. These were “flavone and flavonol biosynthesis” (FH72), “galactose metabolism” (PF48), “starch and sucrose metabolism” (PF48), “ABC transporters” (PF48), and “glycosphingolipid biosynthesis” (PF48), (Table S2). PageRank and diffusion models also targeted these pathways, except for “sphingolipid metabolism” which the hypergeometric test suggested was enriched in PF48 (Table S2).

**Fig. 4:**
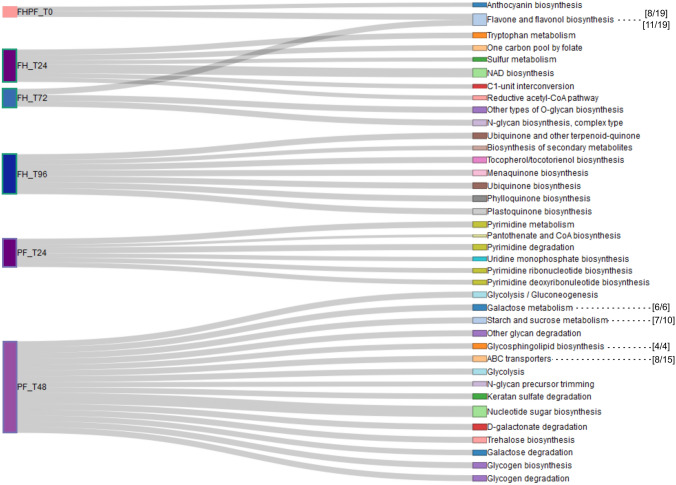
KEGG pathways and modules enriched by explanatory features from wheat lines PF 080719 (PF) and Fundacep Horizonte (FH) at 0, 24, 48, 72 and 96 h post inoculation (hpi) with *P. tritici-repentis* strain BR154. The width of connector lines represents the enrichment significance (−log_10_(*P*-value)) computed with the diffusion model. The dashed lines indicate overrepresented pathways (*P* < 0.05) identified with hypergeometric test. The square brackets represent the number of compounds found in the pathway (first figure) compared to the total number in the pathway (second figure)

We then used a diffusion model to visualise our data (Picart-Armada et al., 2017), and show enrichment in a discrete set of metabolic processes at each genotype/hpi (Fig. 4). At 24 hpi, FH was enriched in the reductive acetyl-CoA pathway, NAD biosynthesis, and tryptophan, sulphur, and one-carbon (C1 unit) metabolism. No significant pathways were enriched in FH at 48 hpi, but at 72 hpi there were shifts in N- and O-glycosylation related pathways and in the biosynthesis of flavonoids. At 96 hpi, *Ptr* infection affected biosynthetic pathways of secondary metabolites and tocopherol. Menaquinone, ubiquinone, phylloquinone, and plastoquinone metabolism were also targeted. As these are components of photosynthetic processes, could reflect the effects of chloroplast targeting by ToxA. In PF, uridine and pyrimidine metabolism as well as pantothenate and CoA biosynthesis were prominent at 24 hpi. By 48 hpi, the metabolic changes were linked to the enrichment of keratan sulphate, glycosphingolipids, nucleotide sugar, trehalose glycogen, starch, sucrose, galactose, and other glycans, metabolism as well as glycolysis and glucogenesis pathways.

**Fig. 5:**
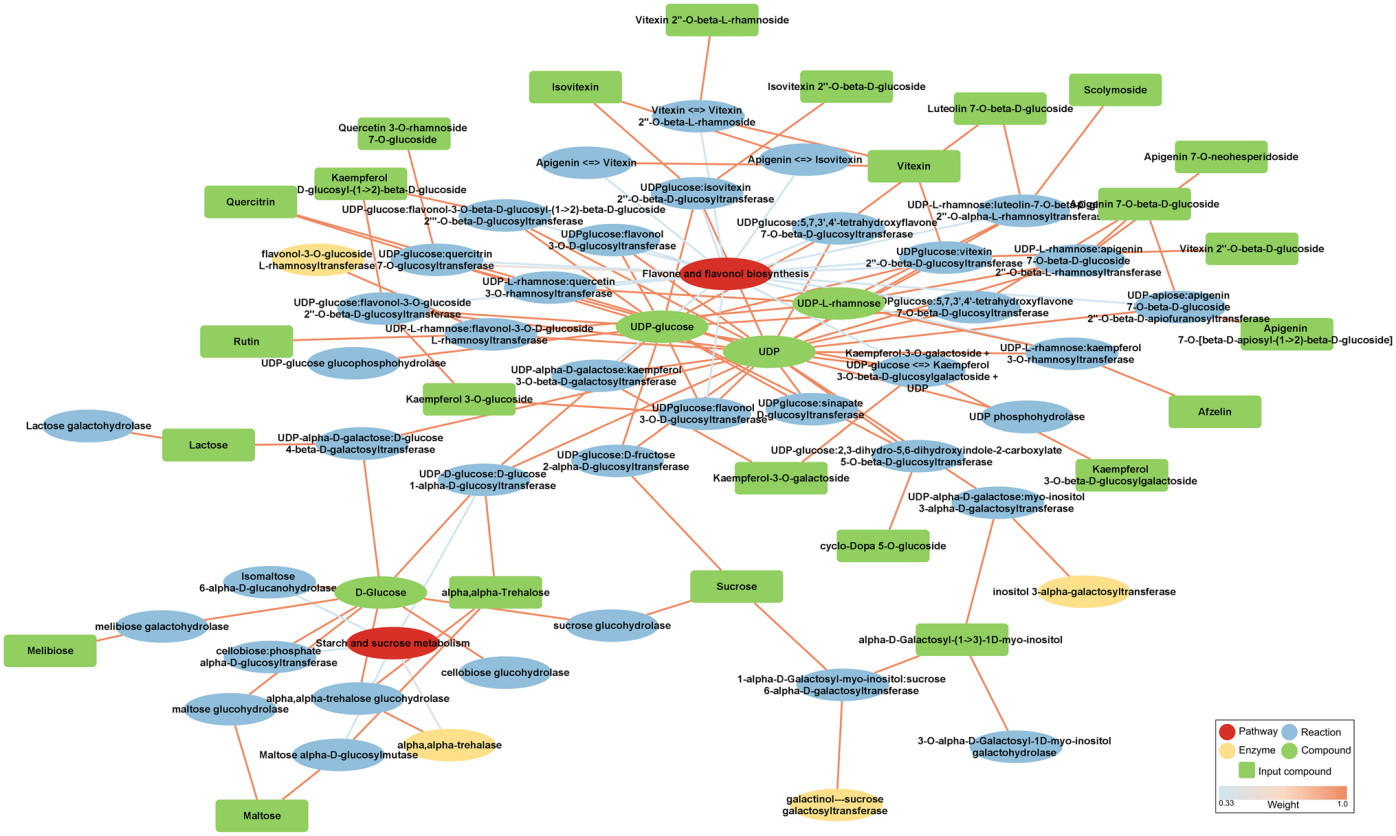
Sub-network showing relevant metabolites, reactions, enzymes, and pathways to wheat-*Ptr* interactome. Explanatory features in the input are highlighted as green squares to indicate the changes leading to enrichment of the presented pathways

To evaluate the metabolic changes in wheat plants induced by *Ptr* infection, an integrative network was constructed using the diffusion enrichment scores. Our network analysis resulted in a sub-graph populated with two key pathways: “flavone and flavonol biosynthesis” and “starch and sucrose metabolism” (Fig. 5). These two pathways are linked via d-glucose, UDP-glucose, UDP, UDP-l-rhamnose. Importantly, the photoassimilate sucrose connects both pathways. This links photosynthetic and bioenergetic changes in the responses of PF and FH to *Ptr.* Furthermore, the network analysis points to three enzymes involved in glycosyltransferase activity (galactinol-sucrose galactosyltransferase, flavonol-3-*O*-glucoside l-rhamnosyltransferase, inositol 3-alpha-galactosyltransferase) and one involved in hydrolase activity, acting on glycosyl bonds (alpha, alpha-trehalase). These enzymes could be key roles in the metabolic changes triggered by *Ptr*.

## Discussion

In this study, we have dissected responses of two wheat genotypes which are agronomical important in Brazil to *Ptr* infection using an untargeted metabolomics approach. PF and FH both proved to be susceptible to *Ptr* at the flag leaf stage, although PF appeared to show some moderate resistance at the seedling stage. In addressing these aspects, we chose to focus on a metabolomic assessments of responses in flag leaves and these have direct impact on grain yield (Bhathal et al., 2003).

Although many facets of the wheat-*Ptr* pathosystem are well established, the metabolic changes occurring with disease development are understudied. These are likely to include changes which are linked to mobilisation of nutrients to the host, an inadequate defence response, the suppression of the defences by *Ptr* and the unregulated effects of symptom development and host cell death (Allwood et al., 2010). Although the disease components are generally specific to a pathosystem, phytopathogens such as *Magnaporthe grisea* are able to induce identical metabolic responses in rice, barley, and *Brachypodium distachyon* (Parker et al., 2009). In our study, whilst there was some overlap in the responses of the two wheat cultivars to *Ptr* (7.9%), most proved to be host specific (Fig. 3b). Before, considering these cultivar-specific responses, those DAMs that were upregulated (53) and downregulated (1) could still represent metabolomic events which are commonly TS susceptibility. Unfortunately, it was not possible to unambiguously identity these metabolites, but they should be assessed in future experiments.

Considering responses in FH following challenge with *Ptr*, we observed major changes in flavonoids (quercitrin, vitexin, and vitexin 2ʺ-*O*-beta-d-glucoside), and coumarin (umbelliferone) (Table S1). These metabolite classes are both derived from the phenylpropanoid pathway and often have direct phytoalexin (anti-microbial) activities or are intermediaries in the production of phytoalexins (Dixon & Paiva, 1995). In wheat, enhanced defences have also been attributed to the antioxidant properties of phenylpropanoid and flavonoid compounds (Gunnaiah & Kushalappa, 2014). The classes of compounds have been linked to resistance to *Fusarium* spp. (Chrpová et al., 2021). For instance, vitexin and quercetin, among other flavonoid compounds, significantly increased with the inoculation of *Fusarium culmorum* in wheat (Buśko et al., 2014). However, our previous transcriptomic-based network analysis has suggested the overexpression of the phenylpropanoid-associated genes phenylalanine ammonia lyase (PAL) and chalcone synthase (CHS), are part of a failed defence to *Ptr* (Ferreira et al., 2022). Similarly, in this study, the accumulation of key flavonoid compounds and umbelliferone would have appeared to be ineffective in controlling *Ptr*. This would imply that phenylpropanoids/flavonoid production is most effective only when part of a wider defence response, possibly co-ordinated by relevant resistance genes (Faris et al., 2013) which were lacking in the *Ptr-*FH interaction. A similar phenomenon could be observed with the tryptophan biosynthetic pathway which was significantly enriched in FH. Tryptophan is a precursor of metabolites with phytoalexin properties, alkaloids, glucosinolates, and auxins (Radwanski & Last, 1995). We identified the toxic alkaloid piperideine (Matsuura & Fett-Neto, 2015) in FH at 24 hpi and glucosinolates in PF at 48 hpi (Table S1). We hypothesise that *Ptr* may be able to overcome the antimicrobial properties of such compounds.

The enrichment analysis also showed significant changes in tocopherol/tocotrienol, phylloquinone, plastoquinone (PQ), ubiquinone (UQ), and “other terpenoid-quinone” in FH at 96 h post *Ptr* infection (Fig. 4). It is relevant that tocopherol (vitamin E) and phylloquinone (vitamin K1) these metabolites, along with PQ, these are chloroplast located and play essential roles in such as photosynthesis, electron transportation, antioxidation, and membrane stability (Havaux, 2020; Munné-Bosch & Alegre, 2010; Swiezewska, 2004). As the wheat plants were challenged with a ToxA-producing strain of *Ptr*, these results likely reflect the effects of this toxin on chloroplasts and reflect the necrotrophic infection strategy of this pathogen. Wider disease effects which may arise from plant cell death may be indicated by shifts in one-carbon (C1) metabolism, biosynthesis of NAD, ubiquinone (UQ), and other terpenoid-quinone in FH. The one-carbon metabolism takes place in the cytosol, peroxisomes, mitochondria, and chloroplast, whereas NAD and UQ pathways are mostly in the mitochondria (Gakière et al., 2018; Hanson & Roje, 2001; Liu & Lu, 2016). Due to the intimate interplay between mitochondria and chloroplasts (Yoshida & Noguchi, 2011), these changes may to represent the effects of ToxA.

Considering specific changes in PF, these included relatively rapid (24 hpi) alterations in the biosynthesis and metabolism of pyrimidine (Fig. 4). This was seen in the rapid change in uridine metabolism which is part of the de novo pyrimidine synthesis pathway. As uridine monophosphate is associated with salvage, phosphotransfer and carbohydrate metabolism, (Zrenner et al., 2006), this could reflect alterations in bioenergetic primary metabolism to feed ultimately defeated defences and/or mobilise nutrients to the pathogen (Bolton et al., 2008). Alterations of biosynthesis and metabolism of pyrimidine pathways have been shown to be an early signalling for PCD (Stasolla et al., 2004) which would be in line with the necrotrophic lifestyle of *Ptr*. If this were the case, it would suggest that PF is particularly susceptible to chloroplast perturbations provoked by ToxA. Pyrimidine nucleotides are heavily involved in the metabolism of sugars (Kafer et al., 2004). Equally inhibition of de novo pyrimidine synthesis, stimulates the compensatory salvage pathway which has been linked to increased levels of uridine nucleotides and the formation of starch from sucrose (Geigenberger et al., 2005). Therefore, the enrichment of galactose, trehalose, starch, and sucrose metabolism pathways seen in PF at 48 hpi could be linked the alterations in pyrimidine metabolism at 24 hpi.

The lowering of sucrose levels in both genotypes likely reflects its mobilisation to pathogen as also seen with *F. graminearum* infections of wheat (Guenther et al., 2009; Hadinezhad & Miller, 2019). As sucrose is the primary photoassimilate in wheat (Takahashi et al., 1998), its reduction is indirect evidence of injuries in the photosynthetic machinery caused by *Ptr*. Furthermore, lower sugar content in flag leaves will likely have a negative effect on yield (Xu-Dong et al., 2003). However, sugars have wider roles in host responses to phytopathogens (Morkunas & Ratajczak, 2014). For instance, trehalose partially induces resistance of wheat to powdery mildew (*Blumeria graminis* f. sp. *tritici*) (Reignault et al., 2001), besides regulating key biological processes such stomatal conductance (Figueroa & Lunn, 2016) which could influence the efficacy of host penetration by the pathogen.

## Conclusions

Our metabolomic characterisation of flag leaves during *Ptr* infection suggested metabolomic changes which whilst distinctive to the two genotypes were apparently consistent in their strategy. In both genotypes, there appeared to be an initiation of defences, particularly those based on phenylpropanoids, which can be assumed to be defeated by *Ptr*. This defensive suppression include the promotion of host cell death, most likely through ToxA effects in our experiments. This study also implies that saccharides play a role in TS disease development as part of wide bioenergetic changes. This illustrates the usefulness of untargeted metabolomics to uncover key information underlying plant–pathogen interactions, as well as to generate testable hypotheses.

### Supplementary Information

Below is the link to the electronic supplementary material.

Supplementary file1 (XLSX 59 KB)

Supplementary file2 (XLSX 116 KB)

## Data Availability

The metabolomics and metadata reported in this paper are available via www.ebi.ac.uk/metabolights/MTBLS7550 study identifier MTBLS7550.

## References

[CR1] Abdullah S, Sehgal SK, Ali S, Liatukas Z, Ittu M, Kaur N (2017). Characterization of *Pyrenophora tritici-repentis* (tan spot of wheat) races in Baltic States and Romania. The Plant Pathology Journal.

[CR2] Abeysekara NS, Friesen TL, Liu Z, McClean PE, Faris JD (2010). Marker development and saturation mapping of the tan spot Ptr ToxB sensitivity locus Tsc2 in hexaploid wheat. The Plant Genome.

[CR3] Ali S, Francl LJ (2003). Population race structure of *Pyrenophora tritici-repentis* prevalent on wheat and noncereal grasses in the great plains. Plant Disease.

[CR4] Aliferis KA, Faubert D, Jabaji S (2014). A metabolic profiling strategy for the dissection of plant defense against fungal pathogens. PLoS ONE.

[CR5] Allwood J, Clarke A, Goodacre R, Mur LAJ (2010). Dual metabolomics: A novel approach to understanding plant-pathogen interactions. Phytochemistry.

[CR6] Allwood JW, Williams A, Uthe H, van Dam NM, Luis LAJ, Grant MR, Pétriacq P (2021). Unravelling plant responses to stress—The importance of targeted and untargeted metabolomics. Metabolites.

[CR7] Anderson JA, Effertz RJ, Faris JD, Franel LJ, Meinhardt SW, Gill BS (1999). Genetic analysis of sensitivity to a *Pyrenophora tritici-repentis* necrosis-inducing toxin in durum and common wheat. Phytopathology.

[CR8] Araus JL, Slafer GA, Reynolds MP, Royo C (2002). Plant breeding and drought in C3 cereals: What should we breed for?. Annals of Botany.

[CR9] Bhathal JSS, Loughman R, Speijers J (2003). Yield reduction in wheat in relation to leaf disease from Yellow (tan) Spot and Septoria Nodorum Blotch. European Journal of Plant Pathology.

[CR10] Bolton MD, Kolmer JA, Xu WW, Garvin DF (2008). *Lr34*-mediated leaf rust resistance in wheat: Transcript profiling reveals a high energetic demand supported by transient recruitment of multiple metabolic pathways. Molecular Plant-Microbe Interactions.

[CR11] Buśko M, Góral T, Ostrowska A, Matysiak A, Walentyn-Góral D, Perkowski J (2014). The effect of *Fusarium* inoculation and fungicide application on concentrations of flavonoids (Apigenin, Kaempferol, Luteolin, Naringenin, Quercetin, Rutin, Vitexin) in winter wheat cultivars. American Journal of Plant Sciences.

[CR12] Carmo-Silva E, Andralojc PJ, Scales JC, Driever SM, Mead A, Lawson T (2017). Phenotyping of field-grown wheat in the UK highlights contribution of light response of photosynthesis and flag leaf longevity to grain yield. Journal of Experimental Botany.

[CR13] Chrpová J, Orsák M, Martinek P, Lachman J, Trávníčková M (2021). Potential role and involvement of antioxidants and other secondary metabolites of wheat in the infection process and resistance to *Fusarium* spp. Agronomy.

[CR14] Chu CG, Chao S, Friesen TL, Faris JD, Zhong S, Xu SS (2010). Identification of novel tan Spot resistance QTLs using an SSR-based linkage map of tetraploid wheat. Molecular Breeding.

[CR15] Chu CG, Friesen TL, Xu SS, Faris JD (2008). Identification of novel tan Spot resistance loci beyond the known host-selective toxin insensitivity genes in wheat. Theoretical and Applied Genetics.

[CR16] Cunha G, Caierão E, Rosa A (2016). Informações Técnicas para Trigo e Triticale-Safra 2016.

[CR17] Dixon RA, Paiva NL (1995). Stress-induced phenylpropanoid metabolism. The Plant Cell.

[CR18] Effertz R, Meinhardt JW, Anderson SW, Jordahl JA, Francl JG (2002). Identification of a chlorosis-inducing toxin from *Pyrenophora tritici-repentis* and the chromosomal location of an insensitivity locus in wheat. Biochemistry and Cell Biology.

[CR19] El Wazziki H, El Yousfi B, Serghat S (2015). Contributions of three upper leaves of wheat, either healthy or inoculated by *Bipolaris sorokiniana*, to yield and yield components. Australian Journal of Crop Science.

[CR20] Evans JR (1983). Nitrogen and photosynthesis in the flag leaf of wheat (*Triticum aestivum* L.). Plant Physiology.

[CR21] Evans LT, Rawson HM (1970). Photosynthesis and respiration by the flag leaf and components of the ear during grain development in wheat. Australian Journal of Biological Sciences.

[CR22] Faris JD, Anderson JA, Francl LJ, Jordahl JG (1996). Chromosomal location of a gene conditioning insensitivity in wheat to a necrosis-inducing culture filtrate from *Pyrenophora tritici-repentis*. Phytopathology.

[CR23] Faris JD, Friesen TL (2005). Identification of quantitative trait loci for race-nonspecific resistance to tan Spot in wheat. Theoretical and Applied Genetics.

[CR24] Faris JD, Liu Z, Xu SS (2013). Genetics of tan Spot resistance in wheat. Theoretical and Applied Genetics.

[CR25] Ferreira, L. C., Santana, F. M., & Mur, L. A. J. (2022). Network analysis can provide insights into the wheat and *Pyrenophora tritici-repentis* interaction. In *Plant biology*.

[CR26] Figueroa CM, Lunn JE (2016). A tale of two sugars: Trehalose 6-phosphate and sucrose. Plant Physiology.

[CR27] Friesen T, Faris J (2004). Molecular mapping of resistance to *Pyrenophora tritici-repentis* race 5 and sensitivity to Ptr ToxB in wheat. Theoretical and Applied Genetics.

[CR28] Gakière B, Hao J, de Bont L, Pétriacq P, Nunes-Nesi A, Fernie AR, Gakiére B (2018). NAD+ biosynthesis and signaling in plants. Critical Reviews in Plant Sciences.

[CR29] Gamba FM, Lamari L (1999). Mendelian inheritance of resistance to tan Spot [*Pyrenophora tritici-repentis*] in selected genotypes of durum wheat (*Triticum turgidum*). Canadian Journal of Plant Pathology.

[CR30] Gamba FM, Strelkov SE, Lamari L (2012). Virulence of *Pyrenophora tritici-repentis* in the Southern Cone region of South America. Canadian Journal of Plant Pathology.

[CR31] Gauthier L, Atanasova-Penichon V, Chéreau S, Richard-Forget F (2015). Metabolomics to decipher the chemical defense of cereals against *Fusarium graminearum* and Deoxynivalenol accumulation. International Journal of Molecular Sciences.

[CR32] Geigenberger P, Regierer B, Nunes-Nesi A, Leisse A, Urbanczyk-Wochniak E, Springer F (2005). Inhibition of *de novo* pyrimidine synthesis in growing potato tubers leads to a compensatory stimulation of the pyrimidine salvage pathway and a subsequent increase in biosynthetic performance. The Plant Cell.

[CR33] Guenther JC, Hallen-Adams HE, Bücking H, Shachar-Hill Y, Trail F (2009). Triacylglyceride metabolism by *Fusarium graminearum* during colonization and sexual development on wheat. Molecular Plant-Microbe Interactions.

[CR34] Gunnaiah R, Kushalappa AC (2014). Metabolomics deciphers the host resistance mechanisms in wheat cultivar Sumai-3, against trichothecene producing and non-producing isolates of *Fusarium graminearum*. Plant Physiology and Biochemistry.

[CR35] Guóth A, Tari I, Gallé Á, Csiszár J, Pécsváradi A, Cseuz L, Erdei L (2009). Comparison of the drought stress responses of tolerant and sensitive wheat cultivars during grain filling: Changes in flag leaf photosynthetic activity, ABA levels, and grain yield. Journal of Plant Growth Regulation.

[CR36] Hadinezhad M, Miller SS (2019). Response to *Fusarium graminearum* infection in the rachis of a resistant and a susceptible wheat genotype. Canadian Journal of Plant Pathology.

[CR37] Hanson AD, Roje S (2001). One-carbon metabolism in higher plants. Annual Review of Plant Physiology and Plant Molecular Biology.

[CR38] Havaux M (2020). Plastoquinone in and beyond photosynthesis. Trends in Plant Science.

[CR39] Hu W, He X, Dreisigacker S, Sansaloni CP, Juliana P, Singh PK (2019). A wheat chromosome 5AL region confers seedling resistance to both tan Spot and Septoria nodorum blotch in two mapping populations. The Crop Journal.

[CR40] Inoue T, Inanaga S, Sugimoto Y, An P, Eneji AE (2004). Effect of drought on ear and flag leaf photosynthesis of two wheat cultivars differing in drought resistance. Photosynthetica.

[CR41] Kafer C, Zhou L, Santoso D, Guirgis A, Weers B, Park S, Thornburg R (2004). Regulation of pyrimidine metabolism in plants. Frontiers in Bioscience.

[CR42] Kanehisa M, Goto S, Sato Y, Furumichi M, Tanabe M (2012). KEGG for integration and interpretation of large-scale molecular data sets. Nucleic Acids Research.

[CR43] Kariyawasam G, Nelson AC, Williams S, Solomon PS, Faris JD, Friesen TL (2023). The necrotrophic pathogen *Parastagonospora nodorum* is a master manipulator of wheat defense. Molecular Plant-Microbe Interactions.

[CR44] Khaliq I, Irshad A, Ahsan M (2008). Awns and flag leaf contribution towards grain yield in spring wheat (*Triticum aestivum* L.). Cereal Research Communications.

[CR45] Kokhmetova AM, Kovalenko NM, Kumarbaeva MT (2020). *Pyrenophora tritici-repentis* population structure in the Republic of Kazakhstan and identification of wheat germplasm resistant to Tan Spot. Vavilov Journal of Genetics and Breeding.

[CR46] Kumar R, Bohra A, Pandey MK, Pandey AK, Kumar A (2017). Metabolomics for plant improvement: Status and prospects. Frontiers in Plant Science.

[CR47] Lamari L, Bernier CC (1989). Evaluation of wheat lines and cultivars to Tan Spot [*Pyrenophora tritici-repentis*] based on lesion type. Canadian Journal of Plant Pathology.

[CR48] Lamari L, Sayoud R, Boulif M, Bernier CC (1995). Identification of a new race in *Pyrenophora tritici-repentis*: Implications for the current pathotype classification system. Canadian Journal of Plant Pathology.

[CR49] Lamari L, Strelkov SE, Yahyaoui A, Orabi J, Smith RB (2003). The Identification of two new races of *Pyrenophora tritici-repentis* from the host center of diversity confirms a one-to-one relationship in Tan Spot of Wheat. Phytopathology.

[CR50] Li HB, Yan W, Liu GR, Wen SM, Liu CJ (2011). Identification and validation of quantitative trait loci conferring tan Spot resistance in the bread wheat variety Ernie. Theoretical and Applied Genetics.

[CR51] Liu M, Lu S (2016). Plastoquinone and ubiquinone in plants: Biosynthesis, physiological function and metabolic engineering. Frontiers in Plant Science.

[CR52] Liu XH, Liang S, Wei YY, Zhu XM, Li L, Liu PP, Zheng QX, Zhou HN, Zhang Y, Mao LJ, Fernandes CM, Del Poeta M, Naqvi NI, Lin FC (2019). Metabolomics analysis identifies sphingolipids as key signaling moieties in appressorium morphogenesis and function in *Magnaporthe oryzae*. mBio.

[CR53] Manning VA, Hamilton SM, Karplus PA, Ciuffetti LM (2008). The Arg-Gly-Asp-containing, solvent-exposed loop of Ptr ToxA is required for internalization. Molecular Plant-Microbe Interactions.

[CR54] Manning VA, Hardison LK, Ciufetti LM (2007). Ptr ToxA interacts with a chloroplast-localized protein. Molecular Reproduction and Development.

[CR55] Maserumule M, Rauwane M, Madala NE, Ncube E, Figlan S (2023). Defence-related metabolic changes in wheat (*Triticum aestivum* L.) seedlings in response to infection by *Puccinia graminis* f. sp. *tritici*. Frontiers in Plant Science.

[CR56] Mashabela MD, Tugizimana F, Steenkamp PA, Piater LA, Dubery IA, Mhlongo MI (2023). Metabolite profiling of susceptible and resistant wheat (*Triticum aestivum*) cultivars responding to *Puccinia striiformis* f. sp. *tritici* infection. BMC Plant Biology.

[CR57] Matsuura H, Fett-Neto A (2015). Plant alkaloids: Main features, toxicity, and mechanisms of action. Plant Toxins.

[CR58] McIntosh, R. A., Yamazaki, Y., Dubcovsky, J., Rogers, J., Morris, C., Appels, R., & Xia, X. C. (2013). Catalogue of gene symbols for wheat. In *12th International Wheat Genetics Symposium, Yokohama, Japan*. Retrieved April 4, 2022.

[CR59] Morkunas I, Ratajczak L (2014). The role of sugar signaling in plant defense responses against fungal pathogens. Acta Physiologiae Plantarum.

[CR60] Munné-Bosch S, Alegre L (2010). The function of tocopherols and tocotrienols in plants. Critical Reviews in Plant Sciences.

[CR61] Parker D, Beckmann M, Zubair H, Enot DP, Caracuel-Rios Z, Overy DP (2009). Metabolomic analysis reveals a common pattern of metabolic re-programming during invasion of three host plant species by *Magnaporthe grisea*. The Plant Journal.

[CR62] Picart-Armada S, Fernández-Albert F, Vinaixa M, Rodríguez MA, Aivio S, Stracker TH (2017). Null diffusion-based enrichment for metabolomics data. PLoS ONE.

[CR63] Picart-Armada S, Fernández-Albert F, Vinaixa M, Yanes O, Perera-Lluna A (2018). FELLA: An R package to enrich metabolomics data. BMC Bioinformatics.

[CR64] Radwanski ER, Last RL (1995). Tryptophan biosynthesis and molecular genetics biochemical and molecular genetics. The Plant Cell.

[CR65] Rees RG, Platz GJ (1989). Effectiveness of incomplete resistance to *Pyrenophora tritici-repentis* in wheat. Australian Journal of Agricultural Research.

[CR66] Reignault P, Cogan A, Muchembled J, Lounes-Hadj Sahraoui A, Durand R, Sancholle M (2001). Trehalose induces resistance to powdery mildew in wheat. New Phytologist.

[CR67] Rosato A, Tenori L, Cascante M, De Atauri Carulla PR, Martins dos Santos VAP, Saccenti E (2018). From correlation to causation: Analysis of metabolomics data using systems biology approaches. Metabolomics.

[CR68] Sarma GN, Manning VA, Ciuffetti LM, Karplus PA (2005). Structure of Ptr ToxA: RGD-containing host selective toxin from *Pyrenophora tritici-repentis*. The Plant Cell.

[CR69] Seybold H, Demetrowitsch TJ, Hassani MA, Szymczak S, Reim E, Haueisen J, Lübbers L, Rühlemann M, Franke A, Schwarz K, Stukenbrock EH (2020). A fungal pathogen induces systemic susceptibility and systemic shifts in wheat metabolome and microbiome composition. Nature Communications.

[CR70] Shannon P, Markiel A, Ozier O, Baliga NS, Wang JT, Ramage D (2003). Cytoscape: A software environment for integrated models of biomolecular interaction Networks. Genome Research.

[CR71] Singh PK, Crossa J, Duveiller E, Singh RP, Djurle A (2016). Association mapping for resistance to tan Spot induced by *Pyrenophora tritici-repentis* race 1 in CIMMYTs historical bread wheat set. Euphytica.

[CR72] Singh PK, Mergoum M, Gonzalez-Hernandez JL, Ali S, Adhikari TB, Kianian SF (2008). Genetics and molecular mapping of resistance to necrosis inducing race 5 of *Pyrenophora tritici-repentis* in tetraploid wheat. Molecular Breeding.

[CR73] Singh PK, Singh RP, Duveiller E, Mergoum M, Adhikari TB, Elias EM (2010). Genetics of wheat-*Pyrenophora tritici-repentis* interactions. Euphytica.

[CR74] Stasolla C, Loukanina N, Yeung EC, Thorpe TA (2004). Alterations in pyrimidine nucleotide metabolism as an early signal during the execution of programmed cell death in tobacco BY-2 cells. Journal of Experimental Botany.

[CR75] Strelkov SE, Lamari L, Ballance GM (1999). Characterization of a host-specific protein toxin (Ptr ToxB) from *Pyrenophora tritici-repentis*. Molecular Plant-Microbe Interactions.

[CR76] Swiezewska E (2004). Ubiquinone and plastoquinone metabolism in plants. Methods in Enzymology.

[CR77] Tadesse W, Hsam SLK, Wenzel G, Zeller FJ (2006). Identification and monosomic analysis of Tan Spot resistance genes in synthetic Wheat lines (*Triticum turgidum* L. × *Aegilops tauschii* Coss.). Crop Science.

[CR78] Tadesse W, Hsam SLK, Zeller FJ (2006). Evaluation of common wheat cultivars for tan Spot resistance and chromosomal location of a resistance gene in the cultivar ‘Salamouni’. Plant Breeding.

[CR79] Takahashi T, Chevalier PM, Rupp RA (1998). Storage and remobilization of soluble carbohydrates after heading in different plant parts of a winter wheat cultivar. Plant Production Science.

[CR80] Weckwerth W (2003). Metabolomics in systems biology. Annual Review of Plant Biology.

[CR81] Xu-Dong W, Zhen-Wen Y, Dong W (2003). Effect of potassium on sucrose content of flag leaves and starch accumulation of kernels in wheat. Chinese Journal of Plant Ecology.

[CR82] Yang H, Luo P (2021). Changes in photosynthesis could provide important insight into the interaction between wheat and fungal pathogens. International Journal of Molecular Sciences.

[CR83] Ye W, Liu T, Zhang W, Li S, Zhu M, Li H, Kong Y, Xu L (2019). Disclosure of the molecular mechanism of wheat leaf Spot disease caused by *Bipolaris sorokiniana* through comparative transcriptome and metabolomics analysis. International Journal of Molecular Sciences.

[CR84] Yoshida K, Noguchi K, Kempken F (2011). Interaction between chloroplasts and mitochondria: activity, function, and regulation of the mitochondrial respiratory system during photosynthesis. Plant mitochondria: Advances in plant biology 1.

[CR85] Zrenner R, Stitt M, Sonnewald U, Boldt R (2006). Pyrimidine and purine biosynthesis and degradation in plants. Annual Review of Plant Biology.

